# Bayesian modeling of the covariance structure for irregular longitudinal data using the partial autocorrelation function

**DOI:** 10.1002/sim.6465

**Published:** 2015-03-12

**Authors:** Li Su, Michael J Daniels

**Affiliations:** a MRC Biostatistics Unit, Cambridge Institute of Public HealthRobinson Way, Cambridge CB2 0SR, U.K.; b Department of Statistics & Data Sciences, Department of Integrative Biology, University of Texas at AustinAustin, TX 78712, U.S.A.

**Keywords:** missing data, non-stationary covariance function, outcome-dependent follow-up, penalized splines, semiparametric covariance function

## Abstract

In long-term follow-up studies, irregular longitudinal data are observed when individuals are assessed repeatedly over time but at uncommon and irregularly spaced time points. Modeling the covariance structure for this type of data is challenging, as it requires specification of a covariance function that is positive definite. Moreover, in certain settings, careful modeling of the covariance structure for irregular longitudinal data can be crucial in order to ensure no bias arises in the mean structure. Two common settings where this occurs are studies with ‘outcome-dependent follow-up’ and studies with ‘ignorable missing data’. ‘Outcome-dependent follow-up’ occurs when individuals with a history of poor health outcomes had more follow-up measurements, and the intervals between the repeated measurements were shorter. When the follow-up time process only depends on previous outcomes, likelihood-based methods can still provide consistent estimates of the regression parameters, given that both the mean and covariance structures of the irregular longitudinal data are correctly specified and no model for the follow-up time process is required. For ‘ignorable missing data’, the missing data mechanism does not need to be specified, but valid likelihood-based inference requires correct specification of the covariance structure. In both cases, flexible modeling approaches for the covariance structure are essential. In this paper, we develop a flexible approach to modeling the covariance structure for irregular continuous longitudinal data using the partial autocorrelation function and the variance function. In particular, we propose semiparametric non-stationary partial autocorrelation function models, which do not suffer from complex positive definiteness restrictions like the autocorrelation function. We describe a Bayesian approach, discuss computational issues, and apply the proposed methods to CD4 count data from a pediatric AIDS clinical trial. © 2015 The Authors. Statistics in Medicine Published by John Wiley & Sons Ltd.

## 1 Introduction

In long-term follow-up studies, irregular longitudinal data are observed when individuals are assessed repeatedly over time but at uncommon and irregularly spaced time points. Modeling the covariance structure for irregular longitudinal data requires the specification of a covariance function. This specification can be challenging due to the need to keep the covariance function positive definite. Moreover, careful modeling of the covariance structure for the irregular longitudinal data can be crucial for valid inferences on the mean structure in certain settings (e.g., outcome-dependent follow-up and ignorable missing data). In such settings, flexible models for the covariance structure are certainly desirable. In this paper, we describe a flexible approach to modeling the covariance structure for irregular continuous longitudinal data. We will start by providing more details on settings where correctly modeling the covariance structure is essential. We then review the relevant literature on covariance modeling and provide details on our motivating example.

### 1.1 Settings where incorrect covariance modeling results in bias in the mean structure

Irregularly measured longitudinal data can be a result of ‘outcome-dependent follow-up’, where individuals with a history of poor health outcomes are being assessed with greater frequency and regularity. Lipsitz *et al*. 1, Fitzmaurice *et al.* 2, and Ryu *et al.* 3 discussed assumptions that the follow-up time process can be ignored when making likelihood-based inferences about the longitudinal outcome process. Basically, when the follow-up time process only depends on previous outcomes, likelihood-based methods can still provide consistent estimates for the regression parameters in the mean structure of the longitudinal outcome process, and no modeling of the follow-up time process is needed. However, this requires the correct specification of the whole joint distribution of the longitudinal measures for each individual, which of course includes the covariance structure. A similar problem is that of ‘ignorable missing data’. As with the ‘outcome-dependent follow-up’ case, the missing data mechanism does not need to be explicitly specified, but proper inference about the mean structure requires correct specification of the covariance structure 4,5.

### 1.2 Modeling the covariance function

Parametric stationary models for the correlation function have been explored in 6. Qian 7 proposed flexible stationary models that allows the correlation function to decay with lag. Pan and MacKenzie 8 proposed flexible models for irregularly spaced observations using the modified Cholesky decomposition 9 of a covariance matrix, but again, the focus is on ‘stationary-type’ models, where the covariance structure depends on the time lag but not on time. More recently, Zhang and Leng 10 proposed similar (‘stationary-type’) models based on a moving average Cholesky factorization.

Parametric non-stationary models for the covariance function have been proposed in 11 and 12. Diggle and Verbyla 13 proposed nonparametric approaches for the covariance function using kernel weighted local linear regression, but there is no guarantee that their estimator results in a positive definite covariance function. Fan *et al.* 14 proposed semiparametric models, allowing for nonparametric estimation of the variance function, but parametric estimation of the correlation function. Yao *et al.* 15 used a functional principal components analysis approach to modeling the non-stationary covariance structure, but their main purpose was to characterize the time trend and variation of the irregular longitudinal data in a functional data setting. Here, instead, we focus on regression settings, where covariate effects are of interest.

In this paper, we develop a flexible approach to modeling the covariance structure of the irregular continuous longitudinal data by focusing on the partial autocorrelation function (PACF). The advantage of modeling using a PACF is that the only restriction to maintain positive definiteness is to ensure its values are in the interval (−1,1). Therefore, in our approach, the positive definiteness obstacle is removed.

The PACF has been well explored for the stationary setting in the time series literature (e.g., 16) and is often used to determine the order of a stationary autoregressive model in the time series models 17 but appears to have not been used for longitudinal data. Parameterizing a correlation matrix using partial autocorrelations has been explored in 18 and 19 and for a spherical parameterization in 20 but only for cases of *common, equally* spaced follow-up times across units. Zimmerman and Nunez-Anton 21 proposed structured (parametric) antedependence models for the correlation matrix based on partial autocorrelations.

To handle irregularly spaced longitudinal data and accommodate non-stationarity in the correlation function, we develop a new class of semiparametric models for the PACF. To our knowledge, such models have not yet been developed. Together with a model for the variance function, our approach offers flexibility in modeling the covariance structure in challenging situations, such as the ‘outcome-dependent follow-up’ and ‘ignorable missing data’ problems described earlier.

### 1.3 Motivating example

This work was motivated by a randomized double-blinded equivalency trial of high-dose (180 mg per square meter body surface area, six times daily) versus low-dose (90 mg) zidovudine (ZDV) for HIV-infected children (Protocol 128 of the AIDS Clinical Trial Group) 22. The study enrolled 426 children who were randomized to receive one of the two doses and scheduled for measurement of CD4 count before enrollment and every 12 weeks up to 5 years. The analysis objective is to compare the treatment-specific longitudinal trajectories of CD4 counts.

**Figure 1:**
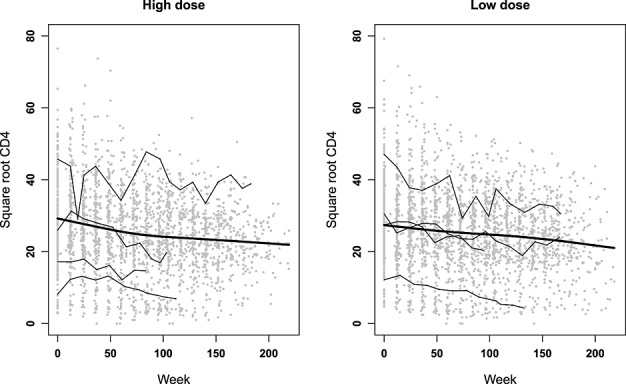
Observed (square root) CD4 count data over time by the dose groups, with local regression fits to the pooled sample (dark lines) and profiles from 4 selected participants in each group highlighted.

**Figure 2:**
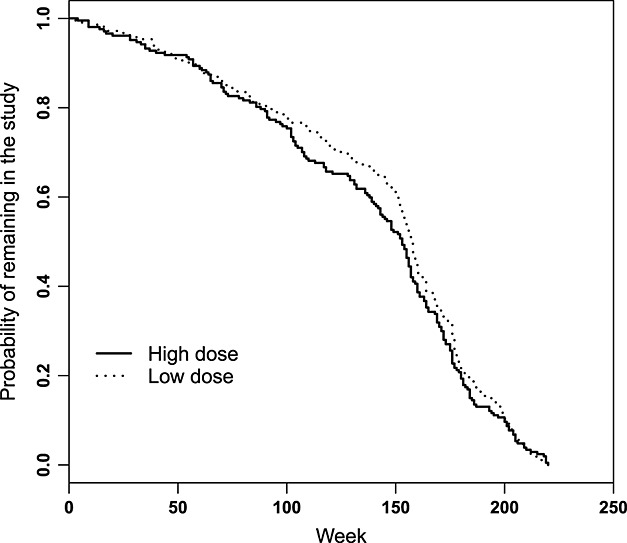
Kaplan-Meier curves for observed dropout times by the dose groups in the AIDS example.

**Figure 3:**
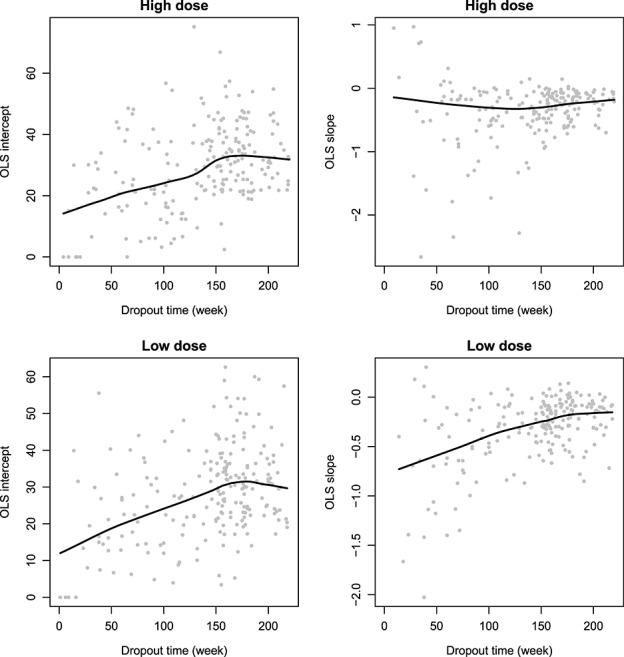
Individual OLS intercepts and slopes of square root CD4 count as functions of the dropout time by the dose groups, with local regression fits highlighted.

However, the actual measurement times were irregular and varied considerably across children. Figure 1 presents the observed CD4 count data over time by the dose groups with local regression fits to the pooled sample and four individual profiles highlighted. Note that a square root transformation is used to reduce the right skewness in these data. The total number of measurements was 4999, while the number of measurements per child varied from 1 to 21. The observed maximum follow-up time was 219 weeks, and there were 214 unique measurement times following enrollment. In addition, only about half of the children completed 3 years of follow-up (Figure 2). Previous analyses 5,23 suggest that the dropout was possibly informative in the sense that children with a more rapid decline in CD4 count were more likely to drop out. Figure 3 presents the estimated individual ordinary least-squares intercepts and slopes of the square root of CD4 counts against the observed dropout times, and it appears that lower intercepts in both dose groups and lower slopes in the low-dose group are associated with early dropout. In Section 4, we will demonstrate how to accommodate the irregular measurement times in modeling the covariance structure of these CD4 data while dealing with informative dropout at the same time.

The remainder of this paper is organized as follows. In Section 2, we formally define partial autocorrelations and the PACF, then describe a flexible class of semiparametric non-stationary models for the covariance function. Section 3 discusses the computational issues and ‘tricks’ that can be used to perform Bayesian (or likelihood) inference efficiently. In Section 4, we apply the proposed methods to the pediatric CD4 count data. We offer conclusions and extensions in Section 5.

## 2 Definitions and models

Suppose that *N* independent individuals are to be followed up intermittently at discrete time *t* = 1,2,…,*T*. Here, *t* could be months, weeks, days or hours (in the AIDS example in Section 4, it is ‘months’). The constant *T* is determined by the potential maximum follow-up time where a longitudinal measurement can be taken in the study; and the value of *T* can be large depending on the chosen time unit. We assume a discrete time Gaussian process for the longitudinal outcome from the *i*th individual (*i* = 1,…,*N*), {*Y*_*i*_(*t*):*t*∈1,…,*T*}, with a mean function *μ*_*i*_(*t*) and a covariance function, Cov{*Y*_*i*_(*t*),*Y*_*i*_(*t*′)}=*σ*_*i*_(*t*)*σ*_*i*_(*t*′)*ρ*_*i*_(*t*,*t*′) (*t*,*t*′∈1,…,*T*), where 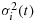 is the variance function and *ρ*_*i*_(*t*,*t*′) = Cor{*Y*_*i*_(*t*),*Y*_*i*_(*t*′)} is the autocorrelation function.

We assume that the mean function *μ*_*i*_(*t*) can be described by a linear model

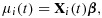
1
where **X**_*i*_(*t*) is a *p*-dimensional covariate process that can include both time-invariant and exogenous time-varying covariates, and ***β*** is the *p* × 1 vector of corresponding regression coefficients. Note that random effects can be added (we will do this in the data analysis in Section 4) or more flexible structures can be specified for the mean function, for example, using semiparametric regression approaches 24. In this paper, however, we focus on developing flexible models for the covariance function Cov{*Y*_*i*_(*t*),*Y*_*i*_(*t*′)} and assume that the mean function can be appropriately modeled by the linear model in 1, possibly with random effects.

At time points 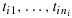, the continuous longitudinal outcome measurements 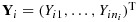 for the *i*th individual are taken. Thus, the covariance matrix of **Y**_*i*_, *Σ*_*i*_ is *n*_*i*_-dimensional. This matrix can be decomposed as ***Σ***_*i*_=**S**_*i*_**R**_*i*_**S**_*i*_, where 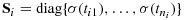 is the diagonal matrix of standard deviations, and **R**_*i*_={*ρ*(*t*_*ik*_,*t*_*il*_)}_*kl*_ (*k*,*l*∈1,…,*n*_*i*_) is the correlation matrix. Note that 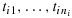 can vary across individuals and be unequally spaced, and in this set-up, irregular longitudinal data are accommodated.

The elements of the correlation matrix, **R**_*i*_, the marginal correlations, can be expressed in terms of the PACF. In this paper, we model the autocorrelation function *ρ*_*i*_(*t*,*t*′) = Cor{*Y*_*i*_(*t*),*Y*_*i*_(*t*′)} by parameterizing the PACF. For the remainder of this section, we drop the subscript *i* and introduce the PACF as well as our semiparametric PACF models.

### 2.1 The partial autocorrelation function

The PACF, π(*t*,*t* + *j*) is defined as

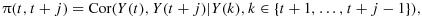

the correlation between *Y*(*t*) and *Y*(*t* + *j*) conditional on the intervening values, {*Y*(*t* + 1),…,*Y*(*t* + *j* − 1)}, where *j* is the time lag. These correlations can also be represented as the correlation of the residuals for *Y*(*t*) and *Y*(*t* + *j*) from regressing each on the intervening values.

In other words, this is the remaining correlation between *Y*(*t*) and *Y*(*t* + *j*) that cannot be explained by all the intervening variables. Therefore, in settings with decaying (serial) correlation, we expect partial autocorrelations to be zero after a certain lag (i.e., with quite a few intervening variables, there is little remaining correlation between *Y*(*t*) and *Y*(*t* + *j*)), unlike marginal correlations.

In addition, the domain of the set of partial autocorrelations induced by the PACF is a [*T*(*T* − 1)/2]−dimensional hypercube, so each can vary independently in (−1,1). This is a major advantage over the autocorrelation function, which is highly restricted. For further intuition on this, we recommend reading about partial correlation vines in the linear algebra literature (references can be found in 18).

In principle, it is easy to move between the PACF, π(*t*,*t* + *j*), and the autocorrelation function, *ρ*(*t*,*t* + *j*) = Cor{*Y*(*t*),*Y*(*t* + *j*)}. In particular, a partial autocorrelation, π(*t*,*t* + *j*), is a function of the correlation matrix corresponding to components (*Y*(*t*),…,*Y*(*t* + *j*)). The following is the expression for computing the marginal correlations from the partial autocorrelations,

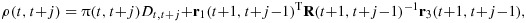

where **R**(*t* + 1,*t* + *j* − 1) is the correlation matrix of the vector {*Y*(*t* + 1),…,*Y*(*t* + *j* − 1)}, **r**_1_(*t* + 1,*t* + *j* − 1)^T^=(*ρ*(*t*,*t* + 1),…,*ρ*(*t*,*t* + *j* − 1)), and **r**_3_(*t* + 1,*t* + *j* − 1)^T^=(*ρ*(*t* + *j*,*t* + 1),…,*ρ*(*t* + *j*,*t* + *j* − 1)). *D*_*t*,*t* + *j*_={1 − **r**_1_(*t* + 1,*t* + *j* − 1)^T^**R**(*t* + 1,*t* + *j* − 1)^−1^**r**_1_(*t* + 1,*t* + *j* − 1)}^1/2^{1 − **r**_3_(*t* + 1,*t* + *j* − 1)^T^**R**(*t* + 1,*t* + *j* − 1)^−1^**r**_3_(*t* + 1,*t* + *j* − 1)}^1/2^is the product of the partial standard deviations 18.

Because *T* can be large in the irregular longitudinal data setting, moving between π(*t*,*t* + *j*) and *ρ*(*t*,*t* + *j*) can be challenging computationally. For example, inverting **R**(*t* + 1,*t* + *j* − 1) can be time-consuming if *j* is large. We discuss solutions to these important computational issues in Section 3.

### 2.2 PACF models

#### 2.2.1 Stationary PACF models

We can construct unrestricted PACF models, using Fisher’s *z*-transform, 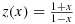 as a link function. To start, we consider a stationary model,

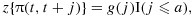
4
where *g* is a monotonically non-increasing function of *j* (time lag), and the indicator function denotes when the lag *j* PACF is zero. The function *g*(*j*) can be formulated as **W**(*j*)***γ***, where **W**(*j*) is a design matrix that is a function of the time lag, *j*; and ***γ*** is a vector of regression coefficients, which does not depend on *t*. This is a stationary PACF model, as π(*t*,*t* + *j*) only depends on the time lag *j*, not *t*.

When *j* > *a*, π(*t*,*t* + *j*) = 0, as we expect that π(*t*,*t* + *j*) will decrease to zero after a certain number of lags, *a*, which is generally expected to not be very large in the serial correlation setting. As a result, this model structure bands the partial autocorrelation matrix, defined as a *T* × *T* matrix with the ones on the main diagonal, and *kl* and *lk*th elements set to π(*k*,*l*) (*k*,*l*∈1,…,*T*), where *a* is the number of bands.

This set-up is important for computations, as it avoids the need to invert large dimensional matrices (when *T* is large) and only requires inversion of at most (*a* + 1)−dimensional matrices when moving between π(*t*,*t* + *j*) and *ρ*(*t*,*t* + *j*) (see Section 3 and Wang and Daniels 25 for further details). Any correlation matrix of the components of {*Y*_1_,…,*Y*_*T*_} will be positive definite under this model.

#### 2.2.2 Semiparametric non-stationary PACF models

We also consider a related non-stationary model,

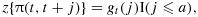
5
where now the function *g* is indexed by time *t* and allows a different rate of decay in partial autocorrelations at different times (thus, non-stationary). The function *g*_*t*_(*j*) can be formulated as **W**(*j*)***γ***(*t*), where **W**(*j*) is a design matrix that is a function of the time lag, *j*; and ***γ***(*t*) is a vector of smooth functions of time, *t*.

In the AIDS example presented in Section 4, we specify a non-stationary PACF model as follows:

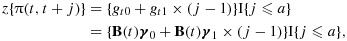
6
where ∀*tg*_*t*1_≤0 and *g*_*t*0_, *g*_*t*1_ are smooth functions of *t* that are modelled nonparametrically using penalized splines with a low-rank thin-plate basis **B**(*t*), that is, *g*_*t*0_=**B**(*t*)***γ***_0_, *g*_*t*1_=**B**(*t*)***γ***_1_ (see details in Section 4). Note that other bases for penalized splines, such as truncated polynomial basis or cubic B-spline basis, can be used. We follow 26 to use the low-rank thin-plate basis because of its better Markov Chain Monte Carlo (MCMC) mixing compared with truncated polynomial basis. In this model, we assume that the partial autocorrelations within band *a* are decaying linearly as a function of *j* by restricting *g*_*t*1_≤0∀*t*. Other more flexible structures in the linear model framework can be specified, for example, by adding a quadratic term of *j*. However, because the partial autocorrelation matrix under our model is banded at *a*, two-dimensional surface estimation for *g*_*t*_(*j*) in 5 is not very practical.

We will choose the number of bands *a* using the Deviance Information Criterion (DIC) 27 in the AIDS example, but it is also possible to put a prior on *a* and obtain its posterior distribution (and integrate over its uncertainty).

### 2.3 Model for the variance function

In some models (e.g., multivariate probit models), the covariance structure is characterized by a correlation matrix (for identifiability) that would be completely specified with the PACF. In our setting with a covariance function, we also need a model for the marginal variance at each time, *σ*^2^(*t*). This could be modeled as a smooth function of time *t* by again using penalized splines on the log scale,

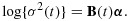
7
In the AIDS example, we use a parametric model for the marginal variance, as the variance over time reveals a simple linear pattern on the log scale (see details in Section 4).

## 3 Overview of posterior computations

For the *i*th individual, we observe the response vector, 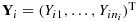, at time points 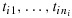. It follows that

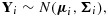
8
where ***μ***_*i*_ is the mean vector that is determined by the model for *μ*_*i*_(*t*) as in 1.

We decompose the marginal covariance matrix ***Σ***_*i*_ such that ***Σ***_*i*_=**S**_*i*_**R**_*i*_**S**_*i*_, where **R**_*i*_ is the marginal correlation matrix, and **S**_*i*_ is a diagonal matrix with marginal standard deviations along the diagonal. **R**_*i*_ is determined by the model for π(*t*,*t* + *j*) as in 4 or 5, and **S**_*i*_ is determined by the model for *σ*^2^(*t*) as in 7.

The corresponding log-likelihood from the *i*th individual is

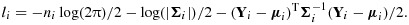
9
Posterior sampling via Gibbs sampling is relatively straightforward. The details of the MCMC algorithm for the AIDS example can be found in the Supplementary Materials.

However, the key to the proposed approach involves efficient ways to compute and invert the covariance matrix for each subject, ***Σ***_*i*_. We now review some results for correlation matrices based on banded partial autocorrelation matrices that make the computation of 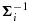 efficient.

Because **R**_*i*_ can be written directly as a function of the marginal correlation function, *ρ*(·,·), we need an efficient algorithm to move between π(·,·) and *ρ*(·,·), as *T* can be quite large. The recursive algorithm proceeds as follows:

1. π(*t*,*t* + 1)→*ρ*(*t*,*t* + 1)
2. *ρ*(*t*,*t* + 1),π(*t*,*t* + 2)→*ρ*(*t*,*t* + 2)
3. *ρ*(*t*,*t* + 1),*ρ*(*t*,*t* + 2),π(*t*,*t* + 3)→*ρ*(*t*,*t* + 3)
  ⋮
  (j) *ρ*(*t*,*t* + 1),…,*ρ*(*t*,*t* + *j* − 1),π(*t*,*t* + *j*)→*ρ*(*t*,*t* + *j*),

where ‘→’ corresponds to the parameters to the left of the arrow being needed to compute the parameters to the right of the arrow based on the formula given in Section 2.1. Moving between π(*t*,*t* + *j*) and *ρ*(*t*,*t* + *j*) also involves inverting a (*j* − 1)-dimensional correlation matrix for the sub-vector (*Y*(*t* + 1),…,*Y*(*t* + *j* − 1)) (*j* = 1,…,*T* − 1) as was seen in Section 2.1. However, the classes of models proposed in Section 2.2 reduces the computational burden by limiting the dimension of matrices that need to be inverted.

Wang and Daniels 25 provide a result for inversion of *a*-band PACF matrices.

**Result 1** 25: Inverting the correlation matrix constructed from an *a*-band partial autocorrelation matrix only requires the inversion of (*a* + 1)-dimensional matrices, and its precision matrix is also an *a*-band matrix.

This result illustrates that these matrix inversions only require inversion of at most (*a* + 1)-dimensional matrices (even for *j* > *a*), which is essential when *T* is large. The expression for inverting these matrices and the proof of this result can be found in the Supplementary Materials to 25.

## 4 Application to the AIDS pediatric trial data

In this section, we use the proposed methods to analyze the AIDS pediatric trial data introduced in Section 1. Recall that the analysis objective is to compare the treatment-specific longitudinal trajectories of CD4 counts. To deal with the informative dropout problem, we use conditional linear models (CLMs) from 28 in the mean structure because dropout occurred in continuous time (Figure 2), and pattern mixture models 29 for discrete dropout times are not suitable here. Similar to pattern mixture models, the joint distribution of the outcome and dropout time in CLMs is factorized as the marginal distribution of the dropout time times the conditional distribution of the outcome given the dropout time. To obtain marginal covariate effects in the mean structure, we do not model the dropout time distribution but use the Rubin’s Bayesian bootstrap 30,31 for averaging over the dropout time distribution.

For the covariance structure, we fit several models to compare their fits to these CD4 data, while the same CLM is assumed for the mean structure. The first is a linear mixed effects model (LMM) with random intercepts and random slopes, which is a simple but often reasonable approach. The marginal correlation structure induced by this model is non-stationary but follows a specific parametric form depending on the covariance matrix of the random effects (see page 133 in 32). Given the large number of unique measurement times and the resulting lack of replications at many times and lags in these CD4 count data, directly estimating an unstructured marginal covariance matrix is not feasible. In addition, we would rather not make strong parametric assumptions about the covariance structure. Thus, we apply our proposed semiparametric PACF model to these CD4 data. In preliminary analyses, we found that the partial autocorrelations were effectively non-zero even at a relatively large number of time lags, which suggests that there was a non-diminishing correlation over time in these data. In addition, we compared model fits for the models with and without random intercepts (to allow for long-term correlation) using DIC (with the random intercepts integrated out) and found that the model with random intercepts provided a better fit to the observed data. We therefore include a random intercept to account for the non-diminishing correlation and use our PACF models to model the serial correlation. We fit both semiparametric and parametric PACF models with different numbers of bands and compare their fits to the observed data using DIC.

Because the maximum of the observed measurement times was 219 weeks, in order to simplify the computations to a more manageable level, we round the actual measurement times to the nearest 4 weeks in the following analyses. Therefore, the maximum follow-up time (i.e., *T*) based on the measurement times is 56. In Section 5, we provide more discussions of the computations when the dimension increases.

### 4.1 Conditional linear model for the mean structure

Let **Y**_*i*_(*t*) represent the square root of CD4 count at time *t* for the *i*th child. In our proposed PACF model, we assume that **Y**_*i*_(*t*) is a discrete-time Gaussian process with a mean function 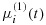,

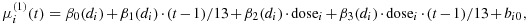
10
where 
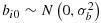
 is a random intercept, dose_*i*_ is an indicator variable for the low-dose group, and *d*_*i*_ is the observed dropout time in weeks (rescaled by taking 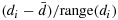, where 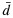 is the sample mean of the observed dropout times). The unit for time *t* is 4 weeks, and *t* = 1,…,56, where *t* = 1 corresponds to enrollment. We scale the time *t* by 13 such that *β*_1_(*d*_*i*_) represents the change rate of CD4 counts over a year, given *d*_*i*_ in the high-dose group.

Following the approach in Wu and Bailey 28, we assume that regression coefficients *β*_0_(*d*_*i*_), *β*_1_(*d*_*i*_), *β*_2_(*d*_*i*_), and *β*_3_(*d*_*i*_) in 10 are functions of the observed dropout time *d*_*i*_. Because Figure 3 shows the local regression fits to the individual ordinary least-squares intercepts and slopes that are not far apart from linear patterns, we assume that *β*_0_(*d*_*i*_) = *θ*_00_+*θ*_01_*d*_*i*_, *β*_1_(*d*_*i*_) = *θ*_10_+*θ*_11_*d*_*i*_, *β*_2_(*d*_*i*_) = *θ*_20_+*θ*_21_*d*_*i*_, and *β*_3_(*d*_*i*_) = *θ*_30_+*θ*_31_*d*_*i*_.

In the LMM, we have the same conditional linear model for the mean as in 10 but add a random slope as follows

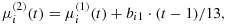
11
where

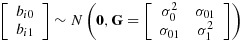

are random effects for the intercept and time slope for the *i*th child.

### 4.2 Models for the covariance structure

For the LMM, we assume that the (residual) covariance function is 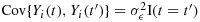.

In the semiparametric PACF model, we assume that

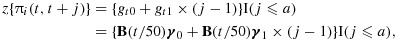

where ∀*tg*_*t*1_≤0, *g*_*t*0_ and *g*_*t*1_ are smooth functions that are modelled nonparametrically using penalized splines with a low-rank thin-plate basis {*B*(*t*)}={1,*t*,|*t* − *ν*_1_|^3^,…,|*t* − *ν*_10_|^3^}, and *ν*_1_<…<*ν*_10_ are 10 equally spaced fixed knots that are set at *t* = 5,10,…,50. For example, the low-rank thin-plate spline representation of *g*_*t*0_ is

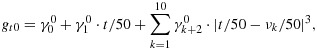
14
where 
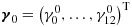
 is a vector of regressioncoefficients. Similarly, we have *g*_*t*1_=**B**(*t*/50)***γ***_1_ and 
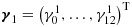
. Note that, to facilitate the numerical computations, we scale *t* by 50 when constructing the penalized spline basis.

For comparison, we also fit a stationary PACF model of the following form:

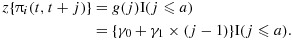

**Figure 4:**
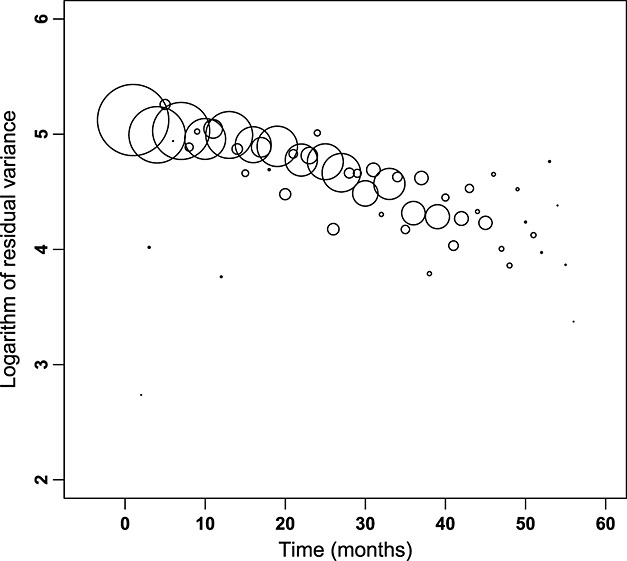
Logarithm of the sample residual variance (from a simple linear model fit to the square root CD4 data) for each time point *t* = 1,…,56 in the AIDS example. The size of the circles is proportional to the number of observations at that time.

For the variance function 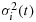, we use a parametric model,

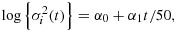
16
because the logarithm of the residual variances (from a simple linear model fit to these data), over time reveals a linear pattern (Figure 4).

### 4.3 Summarizing marginal covariate effects

Following 31, we leave the dropout-time distribution completely unspecified and use the Rubin’s Bayesian bootstrap 30 to obtain the posterior for P(*D* = *d*_*i*_) (see details in 31), where *D* is the dropout time. Basically, at each iteration of the MCMC, we simulate P(*D* = *d*_*i*_) from Dirichlet(1,…,1) for both dose groups. The marginal covariate effects can be approximated by 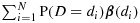, where ***β***(*d*_*i*_) = (*β*_0_(*d*_*i*_),*β*_1_(*d*_*i*_),*β*_2_(*d*_*i*_),*β*_3_(*d*_*i*_))^T^. Details can be found in the Supplementary Materials.

### 4.4 Prior specification and MCMC algorithm

For the mean structure, we specify independent Normal priors *N*(0,10^3^) for *θ*_00_, *θ*_01_, *θ*_10_, *θ*_11_, *θ*_20_, *θ*_21_, *θ*_30_, *θ*_31_.

In the LMM, we use the modified Cholesky decomposition in 33 for modeling the random effect covariance structure, that is, we assume that *b*_*i*1_=*λb*_*i*0_+*e*_*i*_ in 11, where 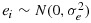. The reparameterization through *λ*, *σ*_0_, and *σ*_*e*_ will guarantee that the covariance matrix of the random effects is positive definite. We assign Uniform(0,20) prior to the standard deviations *σ*_0_ and *σ*_*e*_, and *N*(0,10^3^) prior to *λ*. Note that 
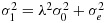
, and we can obtain 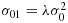. Further, for the (residual) error variance, we assign the prior 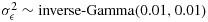.

For both non-stationary and stationary PACF models, we specify independent Normal priors *N*(0,10^3^) for the parameters in the variance function *α*_0_ and *α*_1_ and assign a Uniform(0,20) prior to the random intercept standard deviation *σ*_*b*_.

In addition, for the stationary PACF model, we assign the following priors for *γ*_0_ and *γ*_1_: *γ*_0_∼*N*(0,10^3^), *γ*_1_∼*N*(0,10^3^).

Let 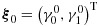, 
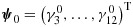
, T_1_=(1,*t*/50), T_2_=(|*t*/50 − *ν*_1_/50|^3^,…,|*t*/50 − *ν*_10_/50|^3^), and ***Ω*** be a 10 × 10 penalty matrix whose (*l*,*k*)th entry is |*ν*_*l*_/50 − *ν*_*k*_/50|^3^. Using the reparameterization 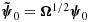 and 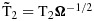, 14 can be rewritten as 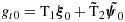. Note that this reparameterization is useful because we can then assign an independent Normal prior to 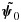 26. Similarly, we can define 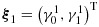, 
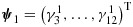
, then 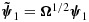 and we have 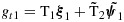.

We assign to ***ξ***_0_, ***ξ***_1_ independent Normal priors with a mean zero and a large variance and to 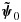 and 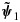 the prior 
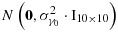
 and 
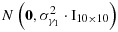
, respectively. Estimating the smoothing parameters 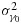 and 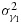 is similar to estimating variance components in Bayesian hierarchical models 34, and the curve estimation by penalized splines can be sensitive to the choice of prior for 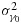 and 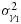. Crainiceanu *et al.* 35 discussed this issue and found that inverse-Gamma priors can be used in practice when certain conditions are met, such that the posterior inference of 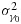 and 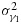 is insensitive to the hyper-parameters in the prior for 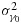 and 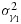. In our application, we use Uniform(0,20) priors for 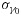 and 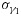.

Because the MCMC algorithm used in the AIDS example involves numerous matrix operations, we decided to use MATLAB (MathWorks, Natick, MA, USA) (instead of R) due to its greater efficiency in matrix operations. The MATLAB code can be run in an open-source alternative QtOctave under Linux. The MATLAB code for fitting the non-stationary PACF models in the AIDS example is available at http://www.mrc-bsu.cam.ac.uk/software/miscellaneous-software/.

Two separate chains were run for each model. Convergence (checked using trace plots) was reached at about 2000 iterations; and pooled samples of 20,000 after convergence were used for inference.

### 4.5 Results

#### 4.5.1 Model selection and assessment

In Table 1, we compare fitted models using DIC based on marginal likelihood with the random effects integrated out. The parameterization used for non-stationary PACF models is *θ*_00_, *θ*_01_, *θ*_10_, *θ*_11_, *θ*_20_, *θ*_21_, *θ*_30_, *θ*_31_***ξ***_0_, ***ξ***_1_, 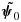, 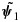, *α*_0_, *α*_1_, 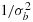. For parametric stationary PACF model, we replace ***ξ***_0_, ***ξ***_1_, 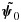, 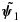 with *γ*_0_ and *γ*_1_. For the LMM, the parameterization is *θ*_01_, *θ*_10_, *θ*_11_, *θ*_20_, *θ*_21_, *θ*_30_, *θ*_31_, **G**^−1^, 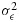.

**Table 1:** DIC values from the fitted models in the AIDS example. 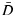 is the posterior mean of the deviance (−2 log*L*), 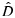 is the deviance by substituting in the posterior means of parameters, 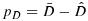 is the effective number of parameters and 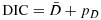.

		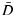	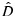	*p*_*D*_	DIC
Linear mixed model		30345	30333	12	30357
Stationary PACF model	Band (*a*)				
	1	30437	30425	12	30449
	2	30187	30175	13	30200
	3	29997	29984	13	30010
	4	29951	29938	13	29964
	5	29955	29942	13	29968
	6	29986	29973	13	29999
Non-stationary PACF model	Band (*a*)				
	1	30405	30387	18	30424
	2	30148	30127	21	30169
	3	29968	29948	20	29988
	4	29911	29890	21	29933
	5	29903	29880	22	**29925**
	6	29922	29899	23	29945

All PACF models except those with one band had smaller DIC values than the LMM fit. The non-stationary PACF model with five bands gives the smallest DIC; therefore, we will present the results based on this model. Note that for each number of bands considered, the non-stationary PACF model results in a smaller DIC than the corresponding stationary PACF model.

To assess the fit of the best model chosen via DIC, we use posterior predictive checks based on replicated observed data as recommended in Daniels *et al.* 36 and a *χ*^2^ discrepancy described in Gelman *et al.* 37. Specifically, the steps are the following:

1. Sample a replicated dropout time, *D*^*rep*^, from the empirical distribution of the observed dropout times.
2. Sample from the empirical distribution of the gap times between irregular measurement times and construct the replicated measurement times up to *D*^*rep*^.
3. Sample a set of responses 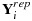 at those measurement times given *D*^*rep*^ and the current posterior sample of the parameters.
4. Repeat Steps 1–3 for all *N* = 421subjects.
5. Compute the *χ*^2^ discrepancy for the replicated data,

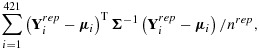
where *n*^*rep*^ is the total number of replicated responses, *μ*_*i*_ is the mean given in 10 with the random effects integrated out, and ***Σ*** is the marginal covariance structure after integrating out the random effects.
6. Compute the *χ*^2^ discrepancy for the observed data.
7. Repeat Steps 1–6 for each posterior sample and compute the posterior predictive probability that the replicated *χ*^2^ statistic is larger than the observed *χ*^2^ statistic.

The posterior probability that the replicated *χ*^2^ statistic is larger than the observed *χ*^2^ statistic is 0.50, which provides no evidence for the lack of fit of the non-stationary PACF model with five bands to the observed data.

#### 4.5.2 Covariance structure

**Figure 5:**
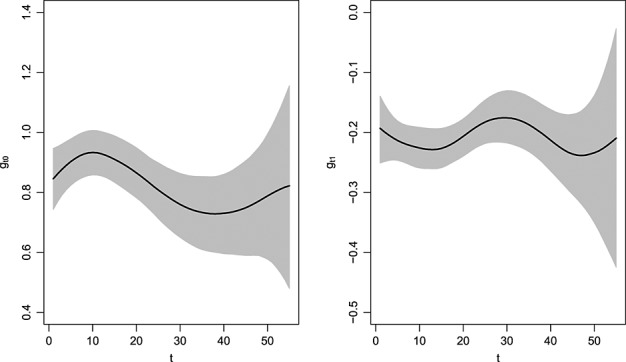
Estimated *g*_*t*0_ and *g*_*t*1_ functions (posterior median and pointwise 95*%* credible band) from the fitted non-stationary 5-band PACF model for the AIDS example.

**Figure 6:**
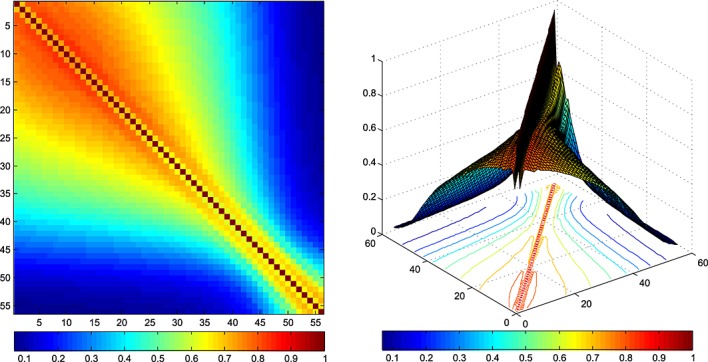
Image plot and surface plot of the estimated 56 × 56 marginal correlation matrix (based on posterior medians of the parameters) from the fitted non-stationary 5-band PACF model for the AIDS example.

Figure 5 shows the estimated functions *g*_*t*0_ and *g*_*t*1_ that determine the non-stationarity of the correlation structure in the fitted five-band non-stationary PACF model. A stationary structure would correspond to both these functions being constant over time. The deviations from stationarity are apparent when *t*∈[0,30]. Figure 6 displays image and surface plots of the correlation structure induced by our PACF model and demonstrates the lack of stationarity as well. In particular, we can see the larger short lag correlations and slower decay at the earlier times versus the later times.

The large estimate for random intercept variance 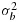 (Table 2) indicates large heterogeneity in terms of the overall CD4 count level across children. For the variance function, there is a significant decrease over time (on the log scale) by noticing that the 95*%* credible interval for the slope, (−2.10,−1.38), does not contain zero (also see Figure 4).

**Table 2:** Posterior medians and 95*%* credible intervals for the parameters of the fitted LMM and non-stationary 5-band PACF model as well as the marginal covariate effects inferred from these models.

	Linear mixed model			5-band PACF model		
	Median	2.5%	97.5%	Median	2.5%	97.5%
*θ*_00_	30.50	28.96	32.02	30.38	28.69	32.07
*θ*_01_	12.87	6.48	19.42	13.12	6.33	19.81
*θ*_10_	−4.97	−5.48	−4.45	−4.82	−5.33	−4.31
*θ*_11_	8.18	5.55	10.80	7.41	5.05	9.82
*θ*_20_	−1.75	−3.86	0.48	−1.56	−3.88	0.79
*θ*_21_	1.26	−8.24	10.63	2.24	−7.17	11.79
*θ*_30_	1.02	0.28	1.76	1.03	0.30	1.76
*θ*_31_	−1.11	−4.86	2.59	−2.21	−5.70	1.18
*σ*_*ϵ*_	3.94	3.86	4.02			
*σ*_0_	11.56	10.80	12.42			
*σ*_01_	−22.62	−27.86	−18.22			
*σ*_1_	3.16	2.89	3.46			
*α*_0_				4.65	4.48	4.81
*α*_1_				−1.76	−2.10	−1.38
*σ*_*b*_				8.01	7.12	8.91

	**Marginal covariate effects**					
	**Linear mixed model**			**5-band PACF model**		
	Median	2.5%	97.5%	Median	2.5%	97.5%
Intercept	30.28	28.67	31.85	30.15	28.42	31.90
Time	−5.11	−5.71	−4.52	−4.95	−5.55	−4.38
Dose	−1.39	−3.62	0.94	−1.19	−3.62	1.23
Interaction	1.23	0.40	2.06	1.21	0.41	2.01

#### 4.5.3 Mean structure

Although the same CLM is applied to both the LMM and the non-stationary five-band PACF model, the parameter estimates in the mean structure differ in these fits (Table 2). For example, the parameter *θ*_11_ quantifies the degree of association of CD4 change rate from baseline to the maximum follow-up with the dropout time in the high-dose group. The large positive estimates from both model fits suggest that later dropouts were associated with a slower decline rate of CD4 count over time. However, in the LMM, this estimated association (8.18 vs. 7.41) is stronger than in the non-stationary five-band PACF model. As a result, the LMM imposes a larger adjustment for selection bias due to dropout in the marginal time slope estimate of the high-dose group (-5.11 vs. -4.95) than the non-stationary PACF model.

On the other hand, *θ*_31_ quantifies the degree of association of the interaction between time and dose effects with the dropout time. Both model fits show no evidence of this association although the point estimates are quite different (-1.11 vs. -2.21). Therefore, we expect that in both model fits, the adjustments for selection bias in marginal time slopes will be similar for both dose groups. Consequently, the marginal interactions between time and dose effects are similar in both model fits (1.23 vs 1.21).

Based on the non-stationary five-band PACF model fit (the best fitting model by DIC), there is a significant difference in the slopes between the two treatments (doses) with the 95*%* credible interval for the difference of (0.41,2.01), indicating that the rate of decline in CD4 counts is less severe under the low-dose arm.

Overall, our analyses of these CD4 data demonstrate that models for the covariance structure can influence the inference for the mean structure under complex scenarios with informative dropout and irregular measurement times.

## 5 Discussion

We have proposed a flexible class of semiparametric, non-stationary models for the covariance structure in a Gaussian process for irregular continuous longitudinal data. The analysis of the CD4 count data showed that modeling covariance structures can impact the inference of the mean structure in complex situations with informative dropout and irregular measurement times. Our models will be useful when careful modeling of the covariance structure is required to ensure valid inference of the mean structure.

In the AIDS data, to make the MCMC algorithm quicker, we aggregated the weekly data to months to change the dimension of *T* from 220 to 56. Note that the issue is not matrix inversions as for an *a*-band PACF, only (*a* + 1)-dimensional matrices need to be inverted. However, as the dimension increases, there are more matrices that need to be inverted to move from the PACF to the autocorrelation function. For example, in our computations, converting a three-band 56 × 56 partial autocorrelation matrix to a marginal correlation matrix took 0.25 s in MATLAB 7.1 (2.59GHz CPU, 32GB RAM on PC). However, to convert a three-band 220 × 220 matrix, it took 40 s. This is less of an issue with more powerful High Performance Computing Clusters, as much of this conversion can be parallelized. Therefore, actual recorded measurement times can be used when applying the proposed PACF models in High Performance Computing Clusters environments.

As with other approaches for dealing with informative dropout, sensitivity analysis is required to assess the unverifiable assumption used in our CLM for the mean structure. In particular, the CLM assumes that the slope before dropout is the same as the slope after dropout; the latter is not identifiable from the observed data. Strategies such as in 31 can be adopted for sensitivity analysis.

The proposed PACF models can be extended to categorical and count data by specifying them for a Gaussian process in the mean function (e.g., 38). For example,

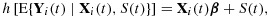

where *h* is a link function, {*S*(*t*)} is a zero-mean Gaussian process with a covariance function Cov(*S*(*t*),*S*(*t* + *j*)) = *σ*(*t*)*σ*(*t* + *j*)*ρ*(*t*,*t* + *j*), and the proposed PACF models can be applied to model *ρ*(·,·). Covariates can be incorporated into the proposed PACF models; we are currently working on this extension.

## Supporting Information

Supporting info item
