# Supplementary material for: Bayesian modeling of the covariance structure for irregular longitudinal data using the partial autocorrelation function
Source: Stat Med. 2015 Mar 12;34(12):2004–18. doi: 10.1002/sim.6465 (PMC4420715; doi:10.1002/sim.6465)
Supplement: Supplementary file 1 [file sim0034-2004-sd1.pdf]

Supplementary Materials to 'Bayesian modelling of the covariance structure for irregular longitudinal data using the partial autocorrelation function'

Li Su[*], Michael J. Daniels[†]

December 7, 2014

Gibbs sampling algorithm for the AIDS example

1. **update** $\boldsymbol{\theta} = (\theta_{00}, \theta_{01}, \theta_{10}, \theta_{11}, \theta_{20}, \theta_{21}, \theta_{30}, \theta_{31})^{\mathrm{T}}$: with prior for $\boldsymbol{\theta} \sim N(\mathbf{0}, c_0 \cdot \mathbf{I})$, the conditional posterior is $N(\boldsymbol{\mu}_\theta, \boldsymbol{\Sigma}_\theta)$ with

$$\begin{aligned} \boldsymbol{\Sigma}_\theta^{-1} &= c_0^{-1} \cdot \mathbf{I} + \sum_{i=1}^{N} \mathbf{X}_i^{\mathrm{T}} \mathbf{S}_i^{-1} \mathbf{R}_i^{-1} \mathbf{S}_i^{-1} \mathbf{X}_i \\ \boldsymbol{\mu}_\theta &= \boldsymbol{\Sigma}_\theta \sum_{i=1}^{N} \mathbf{X}_i^{\mathrm{T}} \mathbf{S}_i^{-1} \mathbf{R}_i^{-1} \mathbf{S}_i^{-1} (\mathbf{Y}_i - \mathbf{Z}_i b_i), \end{aligned}$$

where $\mathbf{Z}_i$ is a $n_i \times 1$ vector of ones, $\mathbf{X}_i = (\mathbf{x}_{i1}, \ldots, \mathbf{x}_{in_i})^{\mathrm{T}}$ is the design matrix in the mean with $\mathbf{x}_{ij} = (1, d_i, t^*_{ij}, d_i \cdot t^*_{ij}, \mathrm{dose}_i, d_i \cdot \mathrm{dose}_i, \mathrm{dose}_i t^*_{ij}, d_i \cdot \mathrm{dose}_i t^*_{ij})^{\mathrm{T}}$ and $t^*_{ij} = (t_{ij} - 1)/13$.

2. **update** b_i: with $b_i \sim N(0, \sigma_b^2)$, the conditional posterior of b_i is $N(\mu_{b_i}, \sigma^2_{b_i})$ with

$$\begin{aligned} \sigma_{b_i}^{-2} &= \sigma_b^{-2} + \mathbf{Z}_i^{\mathrm{T}} \mathbf{S}_i^{-1} \mathbf{R}_i^{-1} \mathbf{S}_i^{-1} \mathbf{Z}_i \\ \mu_{b_i} &= \sigma_{b_i}^2 \mathbf{Z}_i^{\mathrm{T}} \mathbf{S}_i^{-1} \mathbf{R}_i^{-1} \mathbf{S}_i^{-1} (\mathbf{Y}_i - \mathbf{X}_i \boldsymbol{\theta}) \end{aligned}$$

[*] *li.su@mrc-bsu.cam.ac.uk*; MRC Biostatistics Unit, Robinson Way, Cambridge CB2 0SR, UK
[†] *mjdaniels@austin.utexas.edu*; Department of Statistics & Data Sciences, Department of Integrative Biology, University of Texas at Austin, Austin, TX 78712, USA

3. **update** σ_b^2: with $\sigma_b^2 \sim$ Inverse-Gamma(a_1, a_2), the conditional posterior of σ_b^2 is

$$\text{Inverse-Gamma}(a_1 + N/2, a_2 + \sum_{i=i}^{N} b_i^2/2)$$

4. **update** $\tilde{\boldsymbol{\gamma}}_0$, $\tilde{\boldsymbol{\gamma}}_1$: Let $\tilde{\boldsymbol{\gamma}}_0 = (\boldsymbol{\xi}_0^{\mathrm{T}}, \tilde{\boldsymbol{\psi}}_0^{\mathrm{T}})^{\mathrm{T}}$ and $\tilde{\boldsymbol{\gamma}}_1 = (\boldsymbol{\xi}_1^{\mathrm{T}}, \tilde{\boldsymbol{\psi}}_1^{\mathrm{T}})^{\mathrm{T}}$. With prior $\tilde{\boldsymbol{\gamma}}_0 \sim N(\mathbf{0}, \boldsymbol{\Sigma}_{\gamma_0} = \begin{bmatrix} 10^3 \cdot \mathbf{I}_{2\times 2} & \mathbf{0}_{2\times 10} \\ \mathbf{0}_{10\times 2} & \sigma_{\gamma_0}^2 \mathbf{I}_{10\times 10} \end{bmatrix})$, $\tilde{\boldsymbol{\gamma}}_1 \sim N(\mathbf{0}, \boldsymbol{\Sigma}_{\gamma_1} = \begin{bmatrix} 10^3 \cdot \mathbf{I}_{2\times 2} & \mathbf{0}_{2\times 10} \\ \mathbf{0}_{10\times 2} & \sigma_{\gamma_1}^2 \mathbf{I}_{10\times 10} \end{bmatrix})$, we use a random walk Metropolis algorithm to sample from the conditional posterior

$$\begin{aligned} f(\tilde{\boldsymbol{\gamma}}_0, \tilde{\boldsymbol{\gamma}}_1) \;\propto\; & \exp\left\{ -0.5 \sum_{i=1}^{N} (\mathbf{Y}_i - \mathbf{X}_i\boldsymbol{\theta} - \mathbf{Z}_i b_i)^{\mathrm{T}} \mathbf{S}_i^{-1} \mathbf{R}_i^{-1} \mathbf{S}_i^{-1} (\mathbf{Y}_i - \mathbf{X}_i\boldsymbol{\theta} - \mathbf{Z}_i b_i) \right\} \\ & \prod_{i=1}^{N} |\mathbf{R}_i|^{-1/2} \exp(-0.5 \tilde{\boldsymbol{\gamma}}_0^{\mathrm{T}} \boldsymbol{\Sigma}_{\gamma_0}^{-1} \tilde{\boldsymbol{\gamma}}_0 - 0.5 \tilde{\boldsymbol{\gamma}}_1^{\mathrm{T}} \boldsymbol{\Sigma}_{\gamma_1}^{-1} \tilde{\boldsymbol{\gamma}}_1) \end{aligned}$$

with the restriction $g_{t1} \leq 0$. Note that here $\mathbf{R}_i$ needs to be updated accordingly.

5. **update** α_0, α_1: With prior $\alpha_0 \sim N(0, c_0)$, $\alpha_1 \sim N(0, c_0)$, we use a random walk Metropolis algorithm to sample from the conditional posterior

$$\begin{aligned} f(\alpha_0, \alpha_1) \;\propto\; & \exp\left\{ -0.5 \sum_{i=1}^{N} (\mathbf{Y}_i - \mathbf{X}_i\boldsymbol{\theta} - \mathbf{Z}_i b_i)^{\mathrm{T}} \mathbf{S}_i^{-1} \mathbf{R}_i^{-1} \mathbf{S}_i^{-1} (\mathbf{Y}_i - \mathbf{X}_i\boldsymbol{\theta} - \mathbf{Z}_i b_i) \right\} \\ & \prod_{i=1}^{N} |\mathbf{S}_i|^{-1} \exp(-0.5\alpha_0^2/c_0 - 0.5\alpha_1^2/c_0). \end{aligned}$$

Note that here $\mathbf{S}_i$ needs to be updated accordingly.

6. **update** $\sigma_{\gamma_0}^2$: with $\sigma_{\gamma_0}^2 \sim$ Inverse-Gamma(a_1, a_2), the conditional posterior of $\sigma_{\gamma_0}^2$ is

$$\text{Inverse-Gamma}(a_1 + K/2, a_2 + \sum_{i=i}^{N} \tilde{\boldsymbol{\psi}}_0^2/2),$$

where K is the number of knots in the penalized splines and $\tilde{\boldsymbol{\psi}}_1$.

7. **update** $\sigma_{\gamma_1}^2$: with $\sigma_{\gamma_1}^2 \sim$ Inverse-Gamma(a_1, a_2), the conditional posterior of $\sigma_{\gamma_1}^2$ is

$$\text{Inverse-Gamma}(a_1 + K/2, a_2 + \sum_{i=i}^{N} \tilde{\boldsymbol{\psi}}_1^2/2),$$

where K is the number of knots in the penalized splines.

8. **Update** the marginal covariate effects. Sample $P(D = d_i \mid \text{dose}_i = 1)$ and $P(D = d_i \mid \text{dose}_i = 0)$ separately from Dirichlet$(1, \ldots, 1)$. The marginal covariates effects are approximated as follows: the marginal intercept is $\beta_0 = \sum_{i=1}^{N_0} P(D = d_i \mid \text{dose}_i = 0)(\theta_{00} + \theta_{01} d_i)$, the marginal main time effect is $\beta_1 = \sum_{i=1}^{N_0} P(D = d_i \mid \text{dose}_i = 0)(\theta_{10} + \theta_{11} d_i)$, the marginal main dose effect is $\beta_2 = \sum_{i=1}^{N_1} P(D = d_i \mid \text{dose}_i = 1)(\theta_{00} + \theta_{01} d_i + \theta_{20} + \theta_{21} d_i) - \beta_0$ and the marginal interaction bewteen dose and time effects is $\beta_3 = \sum_{i=1}^{N_1} P(D = d_i \mid \text{dose}_i = 1)(\theta_{10} + \theta_{11} d_i + \theta_{30} + \theta_{31} d_i) - \beta_1$, where N_0 and N_1 are sample sizes in the high and low dose groups, respectively.
